# Methodology: workflow for virtual reposition of mandibular condyle fractures

**DOI:** 10.1186/s40902-023-00373-x

**Published:** 2023-01-20

**Authors:** Jan Matschke, Adrian Franke, Olufunmi Franke, Christian Bräuer, Henry Leonhardt

**Affiliations:** Department of Oral and Maxillofacial Surgery, University Hospital “Carl Gustav Carus” Dresden, Fetscherstr. 74, 01307 Dresden, Germany

**Keywords:** Craniomaxillofacial trauma, Mandibular condyle fractures, Osteosynthesis, Virtual repositioning, Patient-specific implant

## Abstract

**Background:**

Even though mandibular condyle fractures have a high clinical relevance, their treatment remains somewhat challenging. Open reduction and internal fixation are difficult due to narrow surgical approaches, poor overview during reduction, and a possible risk of facial nerve damage. In times of technical endeavors in surgery, there is a trend towards the usage of stable CAD-CAM-implants from additive manufacturing or titanium laser sintering. Up until now, there have not been any reports of fracture treatment of the mandibular condyle using this technique.

**Results and conclusion:**

We present a workflow for virtual repositioning of the fractured mandibular condyle, to manufacture patient-specific implants for osteosynthesis with the intention of use of resorbable metal alloys in the future.

## Introduction

Fractures of the mandible comprise approximately 70% of overall facial fractures. In 30% of the cases, the mandibular condyle is fractured. Thus, there is a high clinical relevance in treating these fractures. Current consensus favors open reduction and internal fixation (ORIF) over conservative treatment [1]. For ORIF of mandibular condyle fractures, we currently use a single 3D-rhombic-shaped implant at the Department of Oral and Maxillofacial Surgery, University Hospital “Carl Gustav Carus,” Dresden. So far, we have been able to receive satisfactory results [2].

The surgical treatment of these fractures can be very challenging. The surgical approach is in direct proximity to the facial nerve, which can be damaged leading to devastating consequences for the patients [3]. Small approaches may complicate the ability to inspect and handle the fracture. Even after successful fracture treatment, removal of the implants may be necessary or is at least advised [4]. This possesses even a higher risk of facial nerve injury due to the cicatrized tissue. Resorbable metal implants are already used in the treatment of condylar head fractures of the mandible [5]. One key disadvantage of magnesium-based osteosynthesis implants is their rigid form, as they cannot be bent. After open reduction, an implant must be perfectly aligned to the specific bone structure for a stable internal fixation. Further development in material sciences will provide magnesium-alloy based osteosynthesis plates that are produced by additive manufacturing, to deliver patient-specific implants (PSI). We therefore would like to introduce a workflow for virtual repositioning of mandibular condyle fractures, in order to create PSI that perfectly match the surface of the fractured side. This process is to achieve satisfactory virtual reduction as a precondition for PSI production without the need of further adjustments to osteosynthesis implants. In the future, the need for removal of osteosynthesis material could be avoided by using biodegradable PSI based on magnesium alloys.

## Materials and methods

For virtual repositioning, we used the program BrainLab® software, with the tool Object Manipulation (version 4.0.0.108, BrainLab®, Brainlab AG, Munich, Germany). The virtually repositioned fragments were exported as STL-files. For the display of the exported STL-files, the software MedViso® (MedViso AB, Lund, Sweden) was used. The manufacturing and processing of the displayed model and osteosynthesis plate was done by Anton Hipp GmbH (Fridingen an der Donau, Germany).

## Description of virtual reposition

**Fig. 1:**
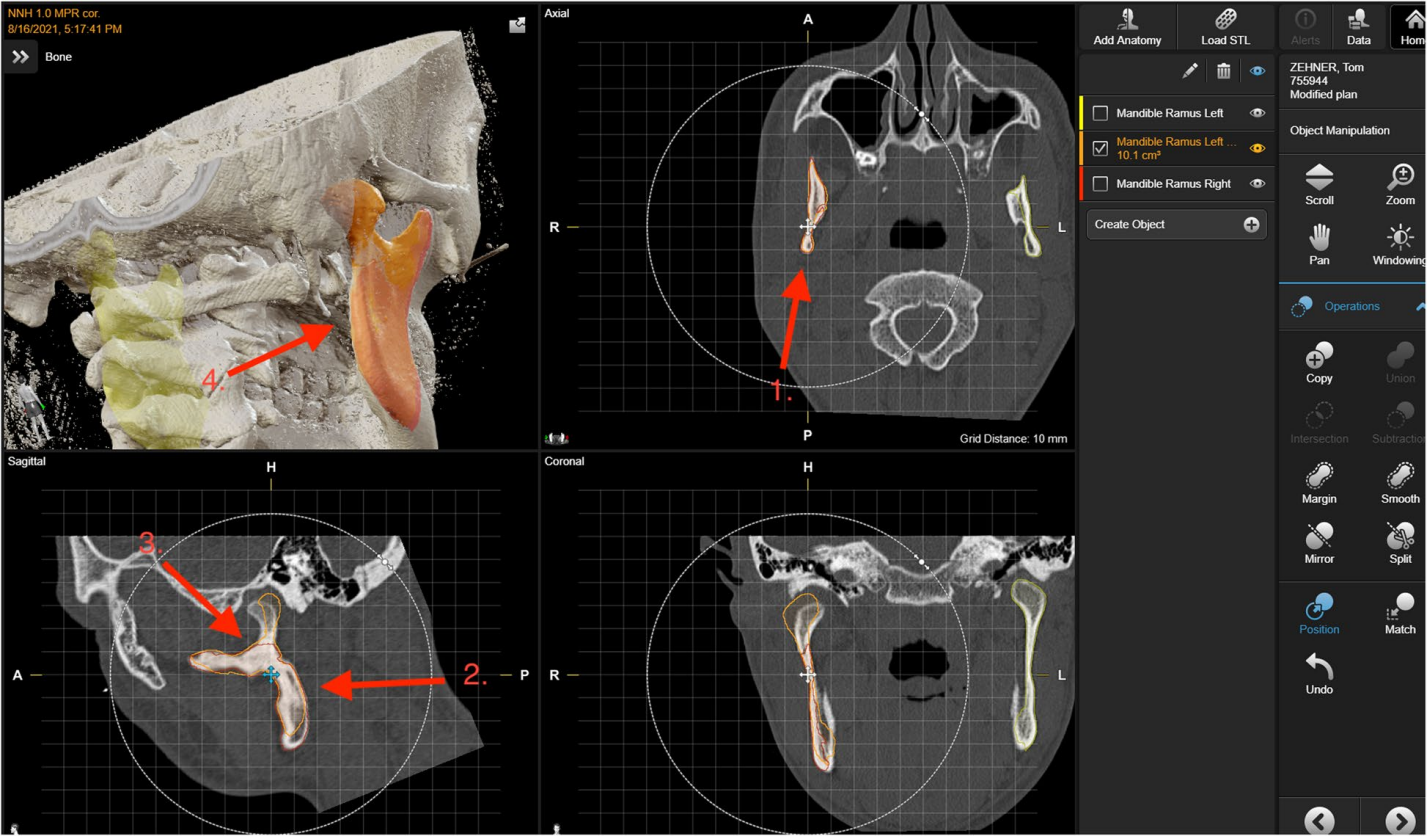
The non-fractured ramus (yellow) is mirrored (orange), afterwards the read edge (1., 2., 4.) and the incisura (3.) must correspond as much as possible. The result is visible at the up left picture, showing a good correspondence and the fracture line on the edge between the mirrored ramus (orange) and the fractured ramus (red). Program used is BrainLab® from Brainlab AG, Munich, Germany

Following facial trauma and admission to the trauma unit, patients normally receive a CT scan of the facial skeleton. If a unilateral mandibular condyle fracture is diagnosed, the introduced workflow can be used for further treatment planning. It is mandatory to use high-resolution CT scans with a maximum thickness of layers of 1 mm for detailed display (depiction) of bony structures of the fracture site. This is a precondition for using the software’s anatomical segmentation tool, as well as manufacture CAD-CAM-based implants. It is important to mention that only patients who suffer from a unilateral condylar fracture and with symmetric mandibular rami can be included. The workflow is not applicable for comminuted condylar fractures, bilateral condylar fractures, and in patients with asymmetric mandibular rami. For the virtual repositioning, the non-fractured, healthy mandibular ramus is firstly segmented by the inbuilt algorithm. The resulting virtual object or segment of the non-fractured ramus needs to be verified and corrected by hand if necessary. The focus needs to be on the posterior edge and the mandibular notch between the muscular process and the mandibular head. Following this, the ramus of the fractured side, without the proximal fragment, has to be segmented. Only then is the proximal fragment segmented. The non-fractured mandibular ramus is mirrored and aligned with the fractured ramus in order to help with the repositioning. To find the correct position, the posterior edge, the mandibular notch, and the lateral curvature in the coronary plane should align as much as possible (Fig. 1).

After successful matching, the fracture line projected onto the segment of the mirrored healthy mandibular ramus indicates where the implant needs to be positioned. In this area, the surface of the segment can be used as a template to produce the PSI (Fig. 1, upper left).

**Fig. 2:**
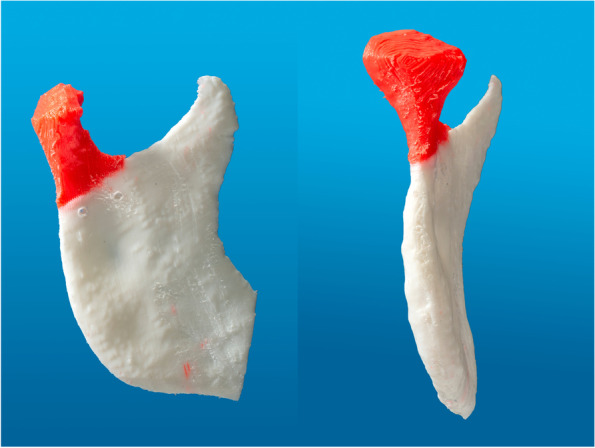
Different views of the right ramus mandibulae (blue) and repositioned right cranial fragment (red); the guide structure is the mirrored ramus mandibulae from the healthy, in this case left, side (white). S, superior; I, inferior; L, left; R, right; P, posterior; A, anterior

**Fig. 3:**
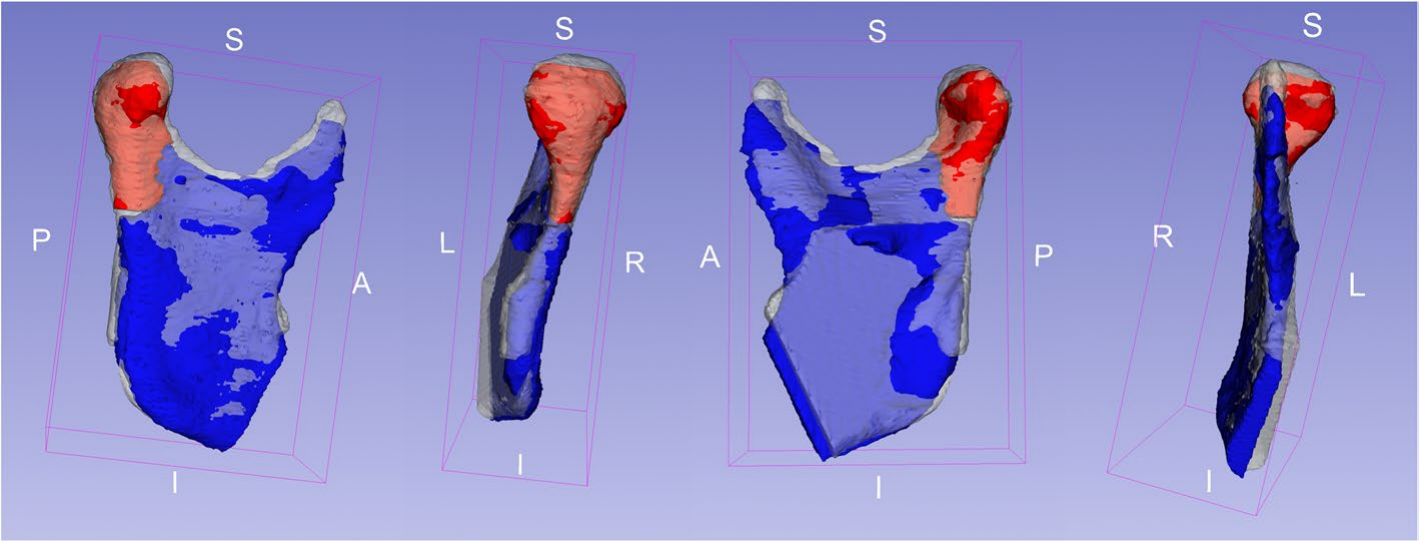
A 3D printed model of the STL-files in different colors

**Fig. 4:**
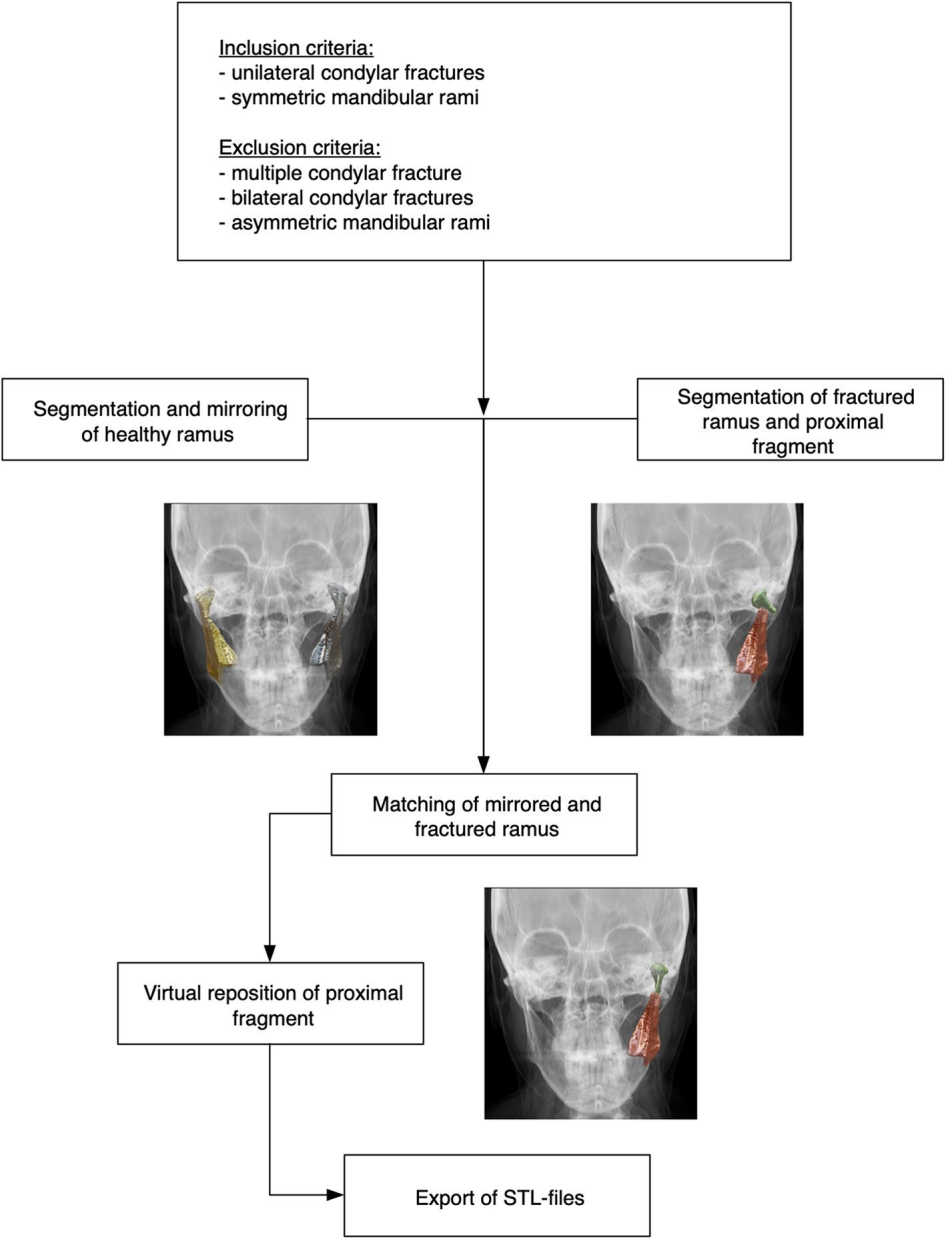
Workflow for the creation of the STL-file

If there is incongruence between the two mandibular rami, a virtual repositioning of the fractured mandibular condyle is necessary. The mirrored healthy ramus is aligned to the fractured ramus as closely as possible. The aforementioned third segment (proximal fragment) can be repositioned using the mirrored ramus as a template structure. The correct position of the ramus and the fragment can be used to produce the PSI (Fig. 2). Subsequently, the segments are exported as STL-Files. In order to highlight the fracture site, the segments can be 3D printed in different colors. The resulting 3D model could be used to customize a standard osteosynthesis plate as an intermediate step to manufacturing the magnesium-based alloy PSI (Fig. 3). All these steps are summarized in our developed workflow (Fig. 4).

## Discussion

In order to avoid complications of implant removal, ongoing development in material sciences continue to provide biodegradable materials for osteosynthesis implants. Current and prospective biodegradable load bearing materials for osteosyntheses are based on magnesium alloys and are non-bendable. Magnesium alloys are already successfully used for the repositioning of condylar head fractures using compression screws [5, 6]. In the future, biodegradable alloys could be used for the treatment of mandibular condyle fractures. Due to the mechanical properties of magnesium-based implants, such plates must optimally match the bony surface of the reduced fracture for exact internal fixation and stable long-term results. As mentioned before, magnesium alloyed plates will not be bendable and therefore must have the correct form and position as no correction can be made during the surgery. This brings a focus on the production of perfectly fitting PSI from additive manufacturing using the help of virtual planning and repositioning. Virtual repositioning is already used in order to provide repositioning devices in surgery [7]. The presented algorithm of fracture segmentation and virtual reduction can assist in facilitating the production of well-fitting PSI. We could find no publication regarding the treatment of condylar neck and base fractures using a PSI. However, the technique of virtual planning and PSI is already used on condylar head fractures [7] and cases of mandibular reconstruction [8].

We believe that the biggest issues lie in firstly placing the PSI in the correct position and secondly, ensuring that there will be no dislocation of the fracture during the fixation process due to a rigid and not perfectly fitting implant. In order to address these problems, clinical studies will commence in the near future.

By using this algorithm and the use of PSI, surgery time can be reduced, leading to shorter narcosis and less manipulation of the tissue. By using biodegradable implants in the future, a second operation to remove the osteosynthesis material may be obsolete. This serves as a great benefit for the patient by avoiding complications as well as further costs for the health care system.

## Conclusion and next steps

Virtual fracture reduction helps in planning of PSI. It can be done easily and quickly using the appropriate software. No clinical tests have been published on this topic, yet. The algorithm is currently under investigation in our clinical studies, and we will report on our results.

## Data Availability

The patient data is not accessible due to privacy concerns.
